# Gestational diabetes mellitus follow-up in Norwegian primary health care: a qualitative study

**DOI:** 10.3399/BJGPO.2021.0104

**Published:** 2022-02-09

**Authors:** Johanne H Toft, Inger Økland, Christina Furskog Risa

**Affiliations:** 1 Department of Obstetrics and Gynecology, Stavanger University Hospital, Stavanger, Norway; 2 Department of Clinical Science, University of Bergen, Bergen, Norway; 3 Department of Caring and Ethics, University of Stavanger, Stavanger, Norway

**Keywords:** diabetes, gestational, qualitative research, primary health care, health promotion, general practice

## Abstract

**Background:**

Women with gestational diabetes mellitus (GDM) have a tenfold increased risk of developing diabetes, and a high risk of recurrent GDM. Endorsing the life-course approach, aiming to prevent disease and promote health across generations, the Norwegian GDM guideline recommends follow-up in primary care after delivery, with information on the increased risks, lifestyle counselling, and annual diabetes screening. Few reports exist on Norwegian women’s experiences of GDM follow-up.

**Aim:**

To elucidate women’s experiences with follow-up of GDM in pregnancy and after delivery, and to explore their attitudes to diabetes risk and motivation for lifestyle changes.

**Design & setting:**

Qualitative study in primary care in the region of Stavanger, Norway.

**Method:**

Semi-structured in-depth interviews were conducted 24–30 months after delivery with 14 women aged 28–44 years, with a history of GDM. Data were analysed thematically.

**Results:**

Most women were satisfied with the follow-up during pregnancy; however, only two women were followed-up according to the guideline after delivery. In most encounters with GPs after delivery, GDM was not mentioned. To continue the healthy lifestyle adopted in pregnancy, awareness of future risk was a motivational factor, and the women asked for tailored information on individual risk and improved support. The main themes emerging from the analysis were as follows: stigma and shame; uncertainty; gaining control and finding balance; and a need for support to sustain change.

**Conclusion:**

Women experienced a lack of support for GDM in Norwegian primary care after delivery. To maintain a healthy lifestyle, women suggested being given tailored information and improved support.

## How this fits in

Despite being at high risk, most women with GDM experience insufficient follow-up after delivery. In Norway, continuity of care is ensured by the GPs being responsible for follow-up before, during, and after pregnancy, as implemented in a new national guideline. However, in the present study, most women experienced a lack of follow-up until 30 months after delivery. Stigma and shame and uncertainty were among the feelings associated with GDM. The participants asked for improved support to sustain change and maintain the healthy lifestyle adopted in pregnancy.

## Introduction

Hyperglycaemia, affecting one in six live births worldwide, is a common medical complication in pregnancy and should be classified as either diabetes mellitus in pregnancy (DIP) or GDM.^1^ In Norway, the prevalence of GDM is now around 6%, after a threefold increase over the past decade.^2^ Among women of non-Scandinavian ethnicity, the prevalence of GDM is higher than in Norwegian women, and the risk of GDM increases with years of residence.^3^

GDM is associated with adverse maternal and fetal outcomes in the short and long term.^4^ Women with prior GDM have a tenfold increased risk of being diagnosed with type two diabetes mellitus (T2DM) later on,^5^ and within 15 years postpartum, one-third of women with GDM have been diagnosed with T2DM.^6^ Moreover, the recurrence rate of GDM is high. For example, in a recent Scandinavian study, the overall recurrence risk of GDM in the second pregnancy was 39%.^7^ As lifestyle intervention reduces the risk of both recurrent GDM and future T2DM, the interconception period is considered as a window of opportunity to improve current and future health of mothers and children.^8^

International guidelines seem to agree on recommending long-term follow-up of women with prior GDM, although the specific tests and schedules vary between countries.^9^ In 2017, a Norwegian GDM guideline was implemented,^10^ implying that follow-up of women with GDM should be done by GPs in primary care, whereas women with poor glycaemic control should be referred to specialist health care. The guideline recommends measurement of HbA1c at 4 months after birth, then annually. Moreover, the GPs should give tailored information about future diabetes risk and offer lifestyle counselling. Most Norwegian citizens are registered with an individual GP, and maternity care is free of charge.

In Norway, introduction of the guideline led to a long-lasting debate about cost-benefit, medicalisation, and in particular, the lack of evidence supporting widespread GDM screening.^11^

Despite diverse guidelines and evidence supporting the effectiveness of early detection of T2DM, long-term follow-up of women with a history of GDM appears challenging worldwide.^12,13^ In England, annual rates of long-term follow-up stayed consistently around 20%,^14^ whereas in the US, rates up to 54% have been reported.^15^ In Australia a national GDM register sends reminders to both mothers and GPs, and the screening rates at 6-week postpartum ranged from 43%–58%, and the annual screening rates were even lower.^16^ In a recent Danish study, women experienced limited initiative from their healthcare providers in supporting them to engage in a healthy lifestyle postpartum.^17^

The life-course approach, which aims to prevent non-communicable diseases (NCDs) such as diabetes and promote health across generations, emphasises pregnancy as an important transition period where there might be unique opportunities to make a positive shift in the trajectory of a generation.^18,19^ Recently, the urgent need to focus on maternal health to prevent NCDs was outlined in a global statement by the International Federation of Gynacology and Obstetrics (FIGO). The importance of preconception counselling, and antenatal and postpartum care was underlined.^20^

To the authors' knowledge, no studies have explored how Norwegian women experience the short- and long-term follow-up of GDM following implementation of the Norwegian guideline. Hence, the aims of this study were to elucidate women’s experiences of GDM follow-up, both in pregnancy and until 30 months after childbirth, and to explore thoughts of future diabetes risk and motivation for lifestyle changes.

## Method

### Study setting

In 2017–2018, 147 nulliparous women aged >25 years with singleton pregnancies participated in a cross-sectional study at Stavanger University Hospital, Norway.^21^ The women had a 75 g oral glucose tolerance test (OGTT) in pregnancy at week 24–28, diagnosing 21 (14%) with GDM. They were informed about the diagnosis, and advised to contact their GP for further follow-up. All women diagnosed with GDM attended a 3-hour workshop, and were offered an ultrasound examination in pregnancy at week 36. According to the Norwegian guideline, women were followed-up in primary care and were referred to secondary health care if glycaemic control was not achieved.

### Sampling and recruitment

**Figure 1.**
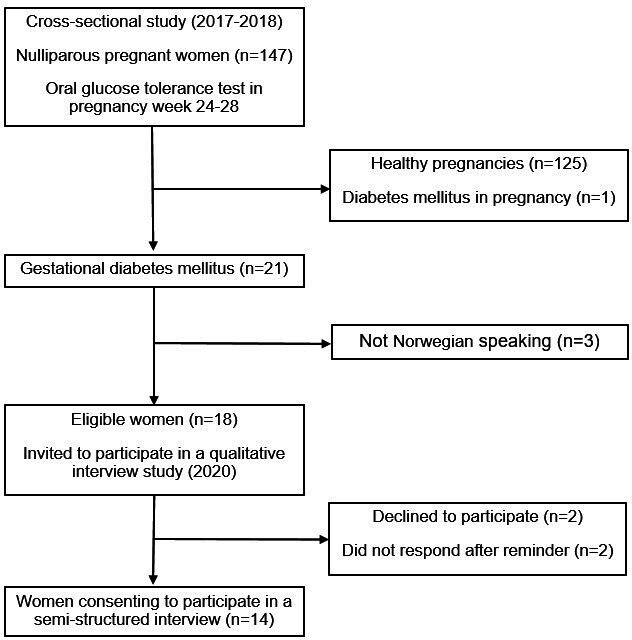
Flowchart of the study population

The qualitative study included all the Norwegian-speaking women, resulting in an eligible study population of 18 women with a history of GDM. To achieve a maximum variety sampling, all 18 women were invited. Information letters describing the aims and method of the study, as well as an informed consent form, were sent in September and October 2020. Women who did not reply within a couple of weeks got a reminder on short message service (SMS). The 14 women consenting to participate signed the informed consent and an appointment for interview was made within 2 weeks. Participants could choose between telephone and face-to-face interviews, and they could choose time and place. Probably owing to the COVID-19 pandemic, they all preferred telephone interviews. A flowchart of the study population is presented in Figure 1.

The current qualitative study was conducted in 2020 and included 14 of the 21 women diagnosed with GDM in the cross-sectional study.^21^

### Data collection

Data were collected using a semi-structured interview guide to explore women’s experiences of the GDM follow-up in pregnancy and after delivery. The interview guide (see Supplementary Appendix S1) consisted of open-ended questions about follow-up, understanding of, and attitude to future diabetes risk, and motivation for lifestyle changes. All interviews were conducted digitally, audio-recorded, and transcribed verbatim by the first author in October 2020, resulting in 99 pages (50 059 words). Each transcript was anonymised and compared with the complete original audio-recording to ensure reliability. The interviews lasted between 19 and 41 minutes, with an average of 30 minutes. All participants received a 50 EUR gift card to acknowledge the authors' gratitude.

### Data analysis

A thematic analysis inspired by Braun and Clarke was conducted on the entire dataset.^22^ An inductive approach was used, where two authors individually read all transcripts several times to gain deeper insight of the material.^23^ Meaningful text relevant to the research questions were highlighted and discussed. Transcripts were then coded line by line by the first author. Accordingly, 205 codes were consecutively sorted into the following four categories, which were in the interview topic guide: experience of being diagnosed with GDM; follow-up; motivation; and future diabetes risk. Next, the codes were collated into broader overarching themes representing repeated patterns across the dataset. Through thorough team discussions a common understanding of the themes was developed. Then a revision and refining of the themes, checking their relation to the coded extracts, was performed by the first author. Finally, after agreement among all authors, an overall interpretation was developed.

Data saturation was achieved during the last two interviews, indicating that no new knowledge relevant to the research questions was obtained. Examples of the analysis from transcript to themes are provided in Table 1. This study is reported in accordance with the Standards for Reporting Qualitative Research (SRQR),^24^ a standard highlighted by the EQUATOR Network (https://www.equator-network.org).

**Table 1.** Example from the data analysis of transforming transcripts to codes and themes

Transcript	Code	Theme
After the initial shock, my stress level decreased. I had to do what was possible, no panic of missing one measurement.Suddenly, gestational diabetes was very serious. Had my GP and I been too laid-back?	Shock getting GDM, stress level decreased gradually.Adequate self-management and follow-up?	Gaining control and finding balanceUncertainty
I was frightened, how could gestational diabetes affect my baby’s health?	Frightened, worried about the baby	Uncertainty

GDM = gestational diabetes mellitus.

## Results

### Demographics

The majority of the 14 women included in the study had a Scandinavian background and almost half had a family history of diabetes. Five women had given birth again, one of these had been diagnosed with recurrent GDM, whereas three women were pregnant. None of the participants had been diagnosed with T2DM. Characteristics of the study population are presented in Table 2.

**Table 2.** Characteristics of the study population (*n* = 14)

Characteristic	Mean (range)	*n* (%)
Age, years	33.7 (28–44)	
Ethnic background		
Scandinavian		11 (79)
Mediterranean or Middle Eastern		3 (21)
Educational level		
Master’s degree		7 (50)
Bachelor’s degree		4 (29)
Student		3 (21)
First-degree relative with diabetes mellitus		6 (43)
Pre-pregnancy BMI (kg/m^2^)^a^	25.4 (20–36)	
Weight gain in pregnancy until OGTT (kg)^a^	10.0 (3–18)	
Insulin use in pregnancy^a^		2 (14)
Interview time-point^b^	27.4 (24–30)	

^a^In first pregnancy. ^b^Months after birth. BMI = body mass index. OGTT = oral glucose tolerance test.

### Main themes

Following the thematic analysis, four main themes emerged: stigma and shame; uncertainty; gaining control and finding balance; and a need for support to sustain change. The themes are discussed in more detail below.

#### Stigma and shame

The majority reported that the GDM diagnosis was surprising, as they did not consider themselves to be at risk. Many described initial feelings of shock, embarrassment, and shame. Some women with obesity and/or family history of diabetes stated that getting GDM was somewhat expected, although it felt tough. Most of the participants associated the diagnosis with unhealthy dietary habits, leading to self-blame for putting the fetus at risk:

*’I felt it was hard, what to say, am I that unhealthy? I did not think so. I actually felt ashamed. Are my eating habits so bad? I felt as a bad mother*.*’* (Participant [P]9)

Several of the participants described situations where they got hurtful comments from others regarding what they ate. The diabetic management made the diagnosis visible to others, and women measured blood glucose in discrete to avoid questions. One of the participants on insulin therapy stated that the feeling of shame increased when she *‘* *could not control* *’* (P3) her blood glucose without insulin, and the multiple injections throughout the day made the diagnosis even more visible to colleagues.

The majority reported a lack of knowledge about GDM. Together with concern for the fetus, this led to anxiety and a call for updated knowledge. Several participants reported difficulties finding reliable information and appreciated the counselling they got from health profesionals. Some of them stated that getting GDM would have been less stigmatic if they had been told about various risk factors for developing GDM, such as family history. Among the participants with a non-Scandinavian background, a common finding was that the feeling of stigma associated with GDM predominantly was related to their ethnic group, resulting in less self-blame at the individual level. Additionally, for some of these women of non-Scandinavian ethnicity, this impaired their motivation for lifestyle changes after birth, as they thought they would develop T2DM anyhow. In contrast, the Scandinavian women associated the diagnosis with unhealthy lifestyle, causing more self-blame at the personal level.

*’I know I will get diabetes in the future anyway. All in my family do*.*’* (P3)

Despite the emotional distress following the diagnosis, concerns for the fetus and wishing to avoid a macrosomic baby seemed to be main motivational factors for lifestyle changes during pregnancy. Other motives were a desire to avoid insulin therapy or induction of labour, or being allowed to stay at the hospital’s low-risk unit.

*’A combination of pressure and fear gave me my motivation. I did it for my own health, but of course, also for my baby’s health*.*’* (P5)

#### Uncertainty

The initial response to the diagnosis was anxiety, partly owing to lack of knowledge and unpredictable implications for the pregnancy. Others became more conscious throughout the pregnancy and after delivery as they learnt more about the increased risks. However, uncertainty affected women’s reactions to the diagnosis, expectations of follow-up, and influenced their thoughts of maternal and fetal risk:

*’During pregnancy I had control because I measured my blood sugar, I knew everything about what to eat and how different food would affect my values. But after pregnancy, I have no idea, how much will it take to develop diabetes in the future?’* (P6)

Whether they actually had GDM was another aspect of uncertainty raised by several participants, as their self-glucose monitorings were within target range, or because of threshold glucose value on the OGTT. Others had become aware of the discussion in the media about the guideline, as well the lack of consensus in GDM-diagnostic criteria among countries. For women experiencing a lack of informational and emotional support, the sense of uncertainty became more manifest.

Overall, women’s glycaemic control was very good, with most values within target range. Nevertheless, induction of labour was decided for one woman because of macrosomia, whereas others were frequently checked owing to fetal growth restriction, cementing their understanding of strict glycaemic control as the most important factor to avoid complications. One woman could not understand why she needed insulin *‘* *to avoid a macrosomic baby* *’* (P3) as her child was small for gestational age:

*’At least, I did not get any explanation why these insulin injections would do anything good for my baby being too small. How insulin would help her, I never got an answer. It was very frustrating taking these injections*.*’* (P3)

Most women presumed they would be diagnosed with GDM in their next pregnancy. A few stated that not getting the diagnosis again felt illogical as they now had a less healthy diet and were more inactive. Moreover, they had not regained pre-pregnancy weight before the second pregnancy.

#### Gaining control and finding balance

Gaining control was a dominant and ongoing theme, involving dietary planning, meals, blood glucose measurements, and clinical follow-ups including ultrasound examinations. Most women reported that self-management, such as incorporating blood glucose measurements in daily life, planning diet, and activity, were most challenging, although achieving glycaemic control also gave mastery and stress relief. However, for several women the burdens of treatment were overwhelming, and two participants described the feeling of having an eating disorder. Others felt obsessed with having a well-controlled diet, with the *‘* *numbers* *’* (several participants) and their blood glucose management dominating their thoughts:

*’I got very upset with the blood sugar measurements. Exercise, eat, and measure. I was obsessed, the measurements should all be good. I talked to my GP about it, and I understood that it could be a big problem for those being too obsessed with this*.*’* (P12)

Some weeks after the GDM diagnosis, many participants realised that finding the right balance in measurements and diet became the most important goal. Others emphasised the emotional support from health professionals to be reassuring.

*’I suddenly realised my life was all about nutrition and table of contents. I got very cautious and strict. I had to remind myself of common sense*.*’* (P14)

#### A need for support to sustain change

Overall, most women contrasted their lack of follow-up after birth with the health care they received for GDM during pregnancy. Most of them stated that the GP did not address the topic of GDM in the encounters after pregnancy. The sense of lack of interest felt like an abandonment, as several requested a need to discuss tailored information regarding their personal risk. Only two women experienced that their GP encouraged them to maintain a healthy lifestyle after pregnancy and had received information about diabetes risk and/or the importance of controlling weight.

*’It has been no talk about GDM. I think when the diagnosis caused all that stress during pregnancy, I was surprised that it has not been mentioned nor followed-up after delivery. I could of course have done more myself, but you know, everyday life continues*.*’* (P14)

Although to a varying degree, most women were aware of the increased diabetes risk and reported that this continuously influenced their lifestyle choices. The majority were concerned about the risk, and thought of this as a motivator to regain pre-pregnancy weight, and maintain a healthy lifestyle for themselves and their family. However, more than half of the participants had gained weight.

*’When I got the diagnosis, I read about the increased diabetes risk, but I am not that worried because I think my food habits are OK and I do exercise; however, by all means, I do think about it and I am aware*.*’* (P17)

Several participants continued to measure blood glucose sporadically after delivery and in the next pregnancy, just to be aware. A few ignored the risks, or thought that their individual risk was low owing to good glycaemic control, a healthy diet, and/or a normal body mass index (BMI).

Nine out of 14 women had measured HbA1c one or more times after their first pregnancy, and all but one stated that this was self-initiated, mostly done when visiting their GP for other reasons. Some reported they were not aware of the recommendation to measure HbA1c, while others had forgotten.

**Figure 2.**
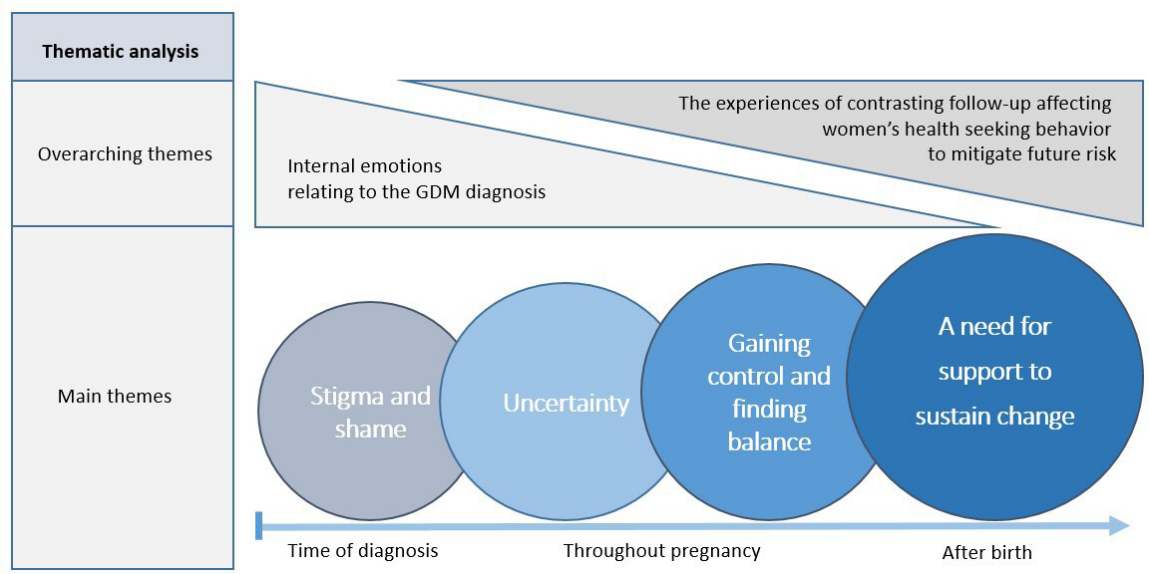
Relationship between overarching themes and main themes along the time course. GDM = gestational diabetes mellitus

There were different opinions among the participants about the preferred time to receive information about diabetes risk; for example, some wanted all information during pregnancy, whereas others stated that the burden of disease and treatment was enough. Moreover, they assumed they would be more receptive after delivery, and several women suggested a GP consultation including HbA1c as part of their maternity care 4–6 months postpartum. A comprehensive understanding of the four main themes described could be included in two broader overarching themes. The first is women’s internal emotions relating to the GDM diagnosis, and the second is the experiences of contrasting follow-up (during and after pregnancy) affecting women’s health-seeking behaviour to mitigate future risk. The relationship between the overarching themes and the main themes along the time course is illustrated in Figure 2, and findings of women’s experiences of GDM follow-up and attitudes to future diabetes risk are summarised in Table 3.

**Table 3.** Study participants' experiences of GDM follow-up, weight development, and attitudes to future diabetes risk (*n* = 14)

Category	*n* (%)
Follow-up in pregnancy^a^	
Good	8 (57)
Middle	3 (21)
Not good	3 (21)
Follow-up after pregnancy^a^	
Good	2 (14)
Middle	2 (14)
Not good	10 (71)
HbA1c measurement after pregnancy	
Participant’s initiative	8 (57)
GP’s initiative	1 (7)
Not measured	5 (36)
Weight development after pregnancy^b^	
Weight gain	8 (57)
Weight loss	6 (43)
The experience with GDM will affect lifestyle and diet in next pregnancy	
Yes	13 (93)
No	1 (7)
Aware of or thinking about future diabetes risk	
Yes	12 (86)
No	2 (14)

^a^Participants were asked to select a response. ^b^Compared with pre-pregnancy weight in first pregnancy. GDM = gestational diabetes mellitus.

## Discussion

### Summary

This study explored women’s experiences of GDM follow-up and attitudes to future risk in Western Norway. The findings indicated that the majority had a positive experience of health care during pregnancy, while most participants stated that they received little or no support for GDM after delivery. Women’s worries about their own and their baby’s health were the major motivational factors for lifestyle changes in pregnancy, and all but one woman noted that their GDM experience would promote a healthy lifestyle in future pregnancies. The majority were aware of being at risk of diabetes and considered this as a motivation to maintain a healthy lifestyle, promoting weight loss after delivery. However, more than half had gained weight. Stigma and shame and uncertainty were among the feelings associated with GDM, and the women asked for improved support to sustain change and maintain a healthy lifestyle.

### Strengths and limitations

This qualitative study has several strengths. First, the participants represent the pregnant population with different ages, various pre-pregnancy BMI, living in both rural and urban parts of the region, and having their follow-up from different GPs. Although the majority were ethnic Norwegians, four women had other ethnic backgrounds. Second, all participants spoke Norwegian fluently, they spoke freely, and gave vivid descriptions of their experiences during the interviews. Third, all interviews were conducted by an experienced resident working at a university hospital’s outpatient clinic for women with complicated pregnancies, who also performed the cross-sectional study from which the participants were recruited.^21^ This background likely improved the quality of the data. Finally, trustworthiness was ensured by involving all authors in the data analysis, a team that was experienced with qualitative studies and thematic analysis.^25,26^

One limitation is that the participants were interviewed 24–30 months after delivery. This might have caused recall bias on participants’ experiences. On the other side, eight of the women were pregnant or had given birth again, giving an opportunity to elucidate their follow-up in the second pregnancy. A semi-structured interview approach was chosen to get a comprehensive understanding of the research questions. This approach is suitable when addressing sensitive topics. All the interviews were conducted by telephone, as preferred by the participants. A limitation with telephone interviews is the miss of facial expressions; however, this does not necessarily influence findings.^27^ Owing to participation in the 2017–2018 study,^21^ detailed information was available on the women's first pregnancy, including background, blood test results, and maternal and fetal outcomes. However, as with other qualitative studies, the present findings rely on self-report, and social desirability bias may have influenced the answers. Finally, the majority of the participants had a master’s or bachelor’s degree, thus, the findings may not be applicable to other socioeconomic groups.

### Comparison with existing literature

Despite the well-documented elevated diabetes risk among women with a history of GDM^5^ and the growing evidence that lifestyle intervention and metformin effectively reduce the long-term risk,^28^ follow-up after delivery appears challenging worldwide.^13,14,29–31^ In the present study, most participants reported that GDM had not been a topic in the encounters with their GPs after delivery, contrary to the recommendations.^10^ As GPs’ experiences of GDM care were not investigated, the findings rely on women’s reports only. In a recent review, women being lost to follow-up and lack of communication between healthcare professionals are barriers mentioned by the providers.^12^ The Norwegian model of care with the GPs being responsible for follow-up before, during, and after pregnancy could facilitate continuity of care for these high-risk women. However, to improve perceived care, women suggest a consultation 4–6 months after birth, including HbA1c, lifestyle counselling, and individualised risk assessment, which is according to the current guideline.^10^

The women in the study got the GDM diagnosis 9–15 months after publication of the guideline. It is well known that guideline implementation and adherence might take several years to fulfil.^32^ However, no difference was observed between women’s satisfaction of follow-up between the start and the end of the study period.

A gap in the quality between recommended and actual care is well documented, also for patients diagnosed with T2DM.^33^ In Norwegian general practice, major gaps in complication screening among patients with diabetes are shown,^34^ and a recent study found large variations in GPs’ performance of care, with patient reminders being one factor associated with better performance.^35^

The Norwegian GDM guideline seems to align with international guidelines in taking the life-course approach. However, regarding GDM, the findings may indicate that some GPs still work within the acute-care paradigm.^36^ To succeed with the life-course perspective a shift in priorities is required. The healthcare systems have traditionally focused on short-term fixes and acute health care. Thus, involvement of policymakers and stakeholders is necessary.^37^ Unfortunately, as observed in other developed countries, Norwegian general practice faces several challenges including growing workload and pressures on funding.^38^

The burden of treatment is described as the workload of health care and its effect on patient functioning and wellbeing.^39^ In accordance with others,^40^ most participants in the present study reported that the burden of GDM was high and medicalisation of pregnancy was apparent. Data analysis revealed ‘uncertainty’ as one of the main themes affecting women’s reactions to the diagnosis, expectations of follow-up, and their attitudes to the increased risk. A recent review evaluating factors affecting uncertainty in high-risk pregnancies concluded that personal, pregnancy-related, demographic, and healthcare-related factors were involved.^41^ Uncertainty was associated with less support and lack of information, and closely tied to appraisal of maternal and fetal risk, as also found in the present study. A further study has reported that uncertainty also affects coping strategies in high-risk pregnant women, and that high levels of uncertainty are associated with emotion-focused rather than problem-focused coping.^42^

The theme ‘gaining control and finding balance’ resonates with others describing the process of being diagnosed and living with GDM as a process from stun to gradual balance.^43^ In a British study, the initial concerns after being diagnosed eased as the women learnt how they could control and manage GDM.^44^

A finding contributing to the burden of disease observed in the present study was women’s awareness of risk and then the following experience of a lack of follow-up, and that they had to request the HbA1c tests themselves. Maybe the motivation for maintaining a healthy lifestyle disappears as the window of opportunity closes? In a recent Scottish study, a lack of aftercare and the need to arrange postnatal testing themselves led some women to question how serious the increased diabetes risk was.^44^

### Implications for research and practice

To reduce the risk of T2DM among women with previous GDM, effective behavioural change interventions are crucial to encourage sustainable change and maintain healthy lifestyles.^45^ A key to successful behavioural change is patient empowerment, where ongoing support helps patients to be responsible for their own health.^46^ In patient empowerment, the health professional's role is to encourage patients to make informed decisions in order to achieve their goals, and providers need to ensure they can support patients to become effective self-managers.

In England, brief, low-cost training of midwives and nurses in healthy conversation skills in a primary care setting was appreciated, and encouraged many women to set goals for behavioural change.^47^ This is in line with the FIGO vision making the best of every contact with women in the reproductive age group.^20^ The FIGO nutrition checklist is another tool for clinicians.^48^ It is approved to be acceptable in routine care, helping to flag-up nutritional at-risk women. Future studies should explore how this could be implemented in a Scandinavian healthcare setting.
